# Gametogenic Development of a Grooved Carpet Shell Clam (*Ruditapes decussatus*, Linnaeus, 1758) Population in the Baldaio Lagoon (N.W. Spain) Amidst Climate Change

**DOI:** 10.3390/ani16030478

**Published:** 2026-02-03

**Authors:** Diana Llamazares, Susana Nóvoa, Justa Ojea, Antonio J. Pazos, M. Luz Pérez-Parallé

**Affiliations:** 1 Acuibiomol Group, Aquatic One Health Research Centre (ARCUS), Department of Biochemistry and Molecular Biology, Universidade de Santiago de Compostela, 15782 Santiago de Compostela, Spain; diana.llamazares.oliveras@usc.es; 2 Centro de Investigacións Mariñas de Galicia (CIMA), Consellería do Mar, Xunta de Galicia, 27700 Ribadeo, Spain; susana.novoa.vazquez@xunta.gal (S.N.); justa.ojea.martinez@xunta.gal (J.O.)

**Keywords:** climate change, grooved carpet shell clam, gametogenic development, Baldaio lagoon

## Abstract

The impact of climate change on marine bivalves, particularly on their reproductive processes, is a current issue of concern. The aim of this study was to investigate how seawater temperatures influenced the gonadal development and overall condition of the grooved carpet shell clam (*Ruditapes decussatus*, Linnaeus, 1758) population in the Baldaio lagoon (N.W. Spain) over the last 20 years. Adult clams were collected, and biometric, histological, and biochemical analyses were performed. Gonadal development phases were assessed, several condition indices were calculated, water temperatures were recorded, and statistical analyses were carried out. Results indicated variations in reproductive timing, including changes in gonadal maturation, an earlier spawning period, and prolonged maturation phases, which contrasted with previous reproductive patterns described for this species. These findings coincided with thermal changes in the lagoon, where mean minimum temperatures increased and maximum temperatures decreased, and the annual thermal range was reduced in comparison with historical data (1998–1999). Biochemical composition and condition indices also reflected variations linked to temperature fluctuations, suggesting that warmer water temperatures may alter energy storage and reproduction. This highlights the importance of continuous environmental monitoring to better understand the effects of climate change on clam populations and to improve management strategies that could help to restore natural *R. decussatus* populations.

## Simple Summary

Climate change affects marine ecosystems, such as coastal lagoons, and therefore influences the lives of the marine invertebrates that inhabit them. In this study, we evaluated the gametogenic development of a grooved carpet shell clam population in the Baldaio lagoon (N.W. Spain). Different parameters, such as biochemical composition, condition indices, and gonadal development, were evaluated throughout the sampling period and compared with the same parameters evaluated 20 years ago. The influence of the seawater temperature of the lagoon on the results is discussed. Compared with previous data, differences in gonadal maturation were found; clams started and finished their reproductive period one month earlier and showed differences in the remaining maturation phases. Understanding these changes is an important issue to protect clam populations and a valuable aid to their management.

## 1. Introduction

The increase in greenhouse gas levels is one of the main problems affecting many aspects of human life, such as weather, agriculture, and food availability, and also has an impact on the ocean. This is mostly due to changes in rainfall patterns and water resources. Higher temperatures and droughts reduce freshwater supplies and accelerate sea level rise [1]. A recent study showed that the Atlantic Ocean has been experiencing constant warming, and its surface temperature has suffered an increasing rate of 0.25 ± 0.03 °C each decade since the 1980s, an increase that is especially notable in the northeastern Atlantic Ocean [2]. The impact on the environment is particularly alarming; higher temperatures are causing damage to forests, coral reefs, and wetlands; this damage includes an increase in the frequency and severity of wildfires that disrupt local ecosystems, the drying out of the wetlands, and the death and bleaching of coral reefs, which affects marine biodiversity and threatens species like fish and marine invertebrates that depend on the reef structures for their habitat and reproduction [3,4]. In 2022 and 2023, marine heatwaves intensified, and their effects reached 60% of the North Atlantic Ocean [2], affecting the entire life cycle of marine organisms. This increasing temperature affects the entire life cycle of marine organisms. As previously stated, this aspect is especially important in the North Atlantic Ocean and along its coast, where a tropicalization of the environment due to the anthropogenic elements affects marine life and impacts the economy [5]. Furthermore, the increasing temperature affects the salinity and density of the water. About 30% of the anthropogenic carbon dioxide has been absorbed by the seabed, causing ocean acidification and changes in the carbonate chemistry, which is unprecedented for 65 million years. This impacts the oxygen and CO_2_ dissolution, which in turn causes changes in pH that have been decreasing 0.017 ± 0.001 units per decade [2]. These changes in the pH are related to modifications of marine currents, metabolic processes, food availability, and, inevitably, the reproduction and growth of marine organisms [1].

Marine invertebrate species, like clams and other bivalve species, are highly influenced by environmental conditions; in fact, they do not regulate their own body temperature, meaning their internal systems are directly affected by the water temperature of the surrounding water [6]. This dependence on external environmental conditions makes them especially vulnerable to changes in the marine ecosystem, such as those caused by climate change. Mannai et al. [7] found that chronic warming stress produces disturbances in both biochemical and physiological parameters in the grooved carpet shell clam (*Ruditapes decussatus*, Linnaeus, 1758). Temperature fluctuations can significantly affect their growth and reproduction, delaying the spawning period as a strategy associated with temperature fluctuations [8], producing abnormal oocytes [9], or changes in feeding behaviours with a negative energy balance that leads to loss of organic weight and mortality [10]. Macho et al. [11] highlighted that heatwaves cause an elevation in the mortality rate of three species of clams, due to the alteration of their capacity to burrow into the sand, which acts as a thermal buffer. Damage to their lipid membrane was detected by Velez et al. [12] in their study on the effects of seawater temperature increase on two clam species, *R. decussatus* and *Ruditapes philiphinarum* (Adams and Reeve, 1850). They found changes in clams’ metabolic response, inducing thermal protection mechanism and alterations in clam behaviour were recorded in their valve closure time.

Other bivalve species like *Ostrea edulis* (Linnaeus, 1758) show a temperature-dependent filter food rate, as it was described by Fabra et al. [13]. When water temperature rises, the solubility of gases like oxygen decreases, leading to low-oxygen or hypoxic conditions, which produce changes in their metabolic processes, such as the metabolic depression caused by a hypoxia episode in juveniles of the little clam *Macoma balthica* (Linnaeus, 1758), described by Jannson et al. [14]. This factor makes it more difficult for organisms such as clams to survive, as they rely on enough oxygen levels to carry out essential physiological processes. Recent studies by Papadopoulos et al. [15] have evaluated the response of R. decussatus to increasing temperatures through the transcription of several genes associated with the antioxidant defence, the anti-apoptotic responses, and the aerobic energy metabolism capacity. These authors have identified three SNPs that were associated with thermal resilience and that could be involved in an adaptive response of this species. Additionally, temperature changes can disrupt their reproductive cycles, affecting their ability to maintain stable populations [9]. Furthermore, it was also shown that ocean acidification affects the susceptibility of bivalves to infections due to lower immunity [16]. Thus, in the environments where temperatures are rising, these species may struggle to survive, leading to a decline in their populations and potentially affecting the entire food chain, as they are a crucial part of marine ecosystems.

The grooved carpet shell clam (*R. decussatus*) is one of the most valuable bivalves as a protein source and one of the most important commercial species along the Galician coast (N.W. Spain). Its geographical distribution extends primarily from the eastern Atlantic Ocean to the Mediterranean coasts, ranging from Norway to Senegal, including the United Kingdom, France, Spain, Portugal, and North Africa. Additionally, it was introduced to the Azores Islands in the 90s [17]. It is an intertidal species that lives buried or partially buried in the marine seabed composed of mud, sand, or fine gravel that provides the individuals shelter and protection from drying in low tide and from high temperatures [18]. Its food supply is provided by phytoplankton, composed of different microalgae species, the production of which is directly linked to the water’s primary production, where they live, which is highly affected by climatic changes [19].

*R. decussatus* is a gonochoric species; male and female individuals cannot be distinguished by sight because the shell shows no visual differences between both sexes. Only when they are fully mature is it possible to observe slight differences in the gonadal tissue inside the visceral mass, although to be certain of the sex, it is necessary to perform smear tests or histological analyses. The reproductive cycle of the grooved carpet shell clam was previously studied in depth [20,21,22,23,24,25]; its spawning period lasts from the end of May until September. During this time, mature adults undergo a few spawning events until the end of the spawning season. In this bivalve species, males and females release sperm and oocytes, respectively, into the water when the appropriate conditions are met; usually the temperature and the pH of the water are the keys that initiate the process, and fertilisation occurs externally [18]. The development of gonadal tissue is directly related to water temperature [26,27], and with increasing ocean temperatures, some differences in the gonadal development of this clam could be expected. In 2004, Ojea et al. [28] studied the seasonal changes in the biochemical composition, weight, body growth, and gonad growth in relation to the gametogenic cycle of *R. decussatus* from a population of Baldaio lagoon (N.W. Spain) and found that they are related to changes in the environmental conditions. The gametogenic cycle of *R. decussatus* showed a resting phase in November–December and gametogenesis in the summer; the spawning period started in June–July [28].

The production of grooved carpet shell clams along the Galician coast has been suffering a decline over the last decade; its production has dropped dramatically, raising concerns among scientists, fishers, and policymakers. In 2013, production levels reached 733 tons, generating a market value of almost 13.5 million euros. By 2023, harvesting data indicated an abrupt fall to 153 tons, with a market value of approximately 6.7 million euros, and in 2024, catches fell even further to just 49 tons and 2.2 million euros in value [29]. This sharp decrease is believed to be influenced by several factors, including environmental changes due to climate change, overexploitation, rainfalls and droughts, predators, exotic invasive species, and habitat degradation [30]. Climate change, through rising sea temperatures, sudden drops in salinity levels due to draughts and ocean acidification, can directly affect the reproduction, survival, and distribution of marine bivalves such as the grooved carpet shell clam. Understanding how these changes impact these species’ reproductive cycles is critical for developing effective conservation and management strategies. This study aims to understand of how sea temperature affects the gametogenic development of the grooved carpet shell clam by comparing the current gonadal development of a population of clams located in the Baldaio lagoon (N.W. Spain) with previous data from approximately 20 years ago by Ojea et al. [28], following the same methodology and procedures, and evaluating the changes that gonadal development may have undergone in this species as a result of environmental changes.

## 2. Materials and Methods

### 2.1. Sample Location and Collection

The selected sampling location was the Baldaio lagoon, on the Atlantic coast of Galicia, in the northwest of Spain. The dimensions of the lagoon are approximately 4000 m in length and between 150 and 350 m in width, with a surface area of 10 km^2^. This lagoon has an average depth between 1 and 2 m, depending on the tide and season of the year. The lagoon is separated from the open sea by a sand barrier, forming a beach barrier lagoon complex, which significantly affects the tidal dynamics within the lagoon. High tide and low tide are attenuated by the sand channel, and there is a mismatch from the open sea due to its special dynamics. This configuration regulates not only the water exchange but also influences temperature and salinity within the lagoon ecosystem. The freshwater input is provided by several small rivers, which directly affect the salinity (especially during low tide) and drainage of the lagoon when the supply of salt water is scarce [31].

The sampling period lasted from March 2023 to January 2025; between 5 and 20 adults of the grooved carpet shell clam were hand-collected per sampling period from natural sandbanks by bivalve harvesters from the same location in the intertidal foreshore of the Baldaio lagoon in approximately monthly intervals. Depending on availability, the exact sample size for each period is reported in Appendix A, Table A1. Due to the special tidal dynamics, logistical problems, and adverse meteorological conditions, some gaps occurred during the sampling period (October, November, and December of 2023 and January and August of 2024). All individuals were dredged up from the sand with a hoe and had the minimum commercial size of 40 mm, as established by local government regulations.

The clams were transported in a refrigerated container to hatchery facilities, where they were placed in 100 L filtered seawater tanks and maintained for 24 h, at the local temperature of the hatchery, in a range of 12–20 °C, depending on the season. The tanks had constant aeration and an open water flow of approximately 100 L per hour to allow the clams to purge their stomach and hepatopancreas and remove any residual sand from their tissues.

Methodology and data collection were carried out following the same procedures in both current and previous studies [28].

### 2.2. Biometric Measurements

For each specimen, external measurements were recorded using a digital calliper with a precision of ±0.01 mm (Mitutoyo IP67 digital caliper, Mitutoyo Corporation, Kawasaki, Kanagawa, Japan). The following dimensions were registered: total shell length (TSL), measured along the anterior–posterior axis; total shell width (TSW), which represents dorsal–ventral size; and total shell thickness (TST), which refers to lateral thickness.

Weight measurements were recorded for each sample using an analytical balance with ±0.01 g precision (RADWAG PS 4500.R2 analytical balance, RADWAG, Radom, Poland). These included total fresh body weight (FBW), including shell; shell weight (SW), only shell weight without visceral mass; fresh soft tissue weight (STW), excluding the shell and gonadal-hepatopancreas tissue weight (GW). Due to the difficulty in separating the gonadal tissue from the visceral mass or hepatopancreas, both tissues were considered as a single unit for weighing in order to avoid potential errors during dissection.

### 2.3. Condition Indices

Biometric measurements were used to calculate the monthly means and several condition indices commonly applied in bivalve studies to assess the physiological state of the clams during the study period. These indices are a useful tool for evaluating reproductive status, energy reserves, and overall health of the clams.

- CI: The condition index was calculated as the ratio between the soft tissue weight and the total weight expressed as a percentage. This index serves as an indicator of the clam’s nutritional and reproductive condition [32].
  (1) $$CI=\left(\frac{STW}{FBW}\right)*100$$
- BCI: Body condition index derived from DCI (dry condition index, defined as the dry weight of the soft body tissue relative to the dry weight of the shell expressed as a percentage). In our case, due to logistical constraints, wet tissue weight was used instead of dry weight, and the index was adapted accordingly, being calculated as a relation between soft tissue weight and shell weight expressed as a percentage [33].
  (2) $$BCI=\left(\frac{STW}{SW}\right)*100$$
- GCI: The gonadal condition index relates gonadal-visceral mass weight with total body weight (excluding the shell) expressed as a percentage; it provides an idea of the gonadal development stage [34].
  (3) $$GCI=\left(\frac{GW}{STW}\right)*100$$

### 2.4. Laboratory Processing and Histology

After measurements, clams were freshly dissected, and tissues were processed using traditional histological techniques [35]. The tissue of the gonad-visceral mass was separated from the rest of the flesh using a scalpel and then longitudinally divided into two parts. One-half was used for a tissue smear on a glass slide for a fresh microscopic examination and then was stored in a sample container and frozen for later biochemical analysis of the gonads. The other half was stored in a biopsy cassette and fixed in aqueous Davidson’s fixing mixture for 24–48 h. After fixation, samples were stored in 70% alcohol until further processing. The cassettes with samples were processed using an automatic benchtop tissue processor (Leica TP1020 Automatic Benchtop Tissue Processor, Leica Biosystems, Barcelona, Spain), where they went through a multi-phase process. The first step was dehydration using increasing alcohol concentration; then they were clarified in xylene, and finally embedded in paraffin at 60°, and hardened samples were used for the formation of paraffin blocks. Using a microtome (MICROM HM 340 E, Microm UK Limited, Rotherham, UK), thin serial sections of 5 µm were cut and placed on glass slides; then they were stained with the haematoxylin-eosin standard staining method. Finally, the slides were mounted using a mounting solution (ClearVue™ Mountant XYL, Epredia™, Kalamazoo, MI, USA), following traditional histological methodology. Then slides were processed and visualised using an optical microscope (Leica DM750 light microscope, Leica Microsystems, Wetzlar, Germany), and photos were taken with a Leica ICC 50 W digital camera and processed and processed with software Leica Application Suite (LAS version 4.12.0, Leica Microsystems, Heerbrugg, Switzerland).

### 2.5. Gonadal Histological Examination

**Figure A1:**
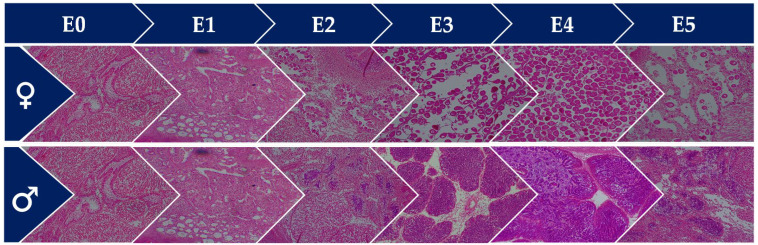
Gonadal qualitative scale of development stages of *Ruditapes decussatus*. (E0: sexual resting stage; E1: onset of gametogenesis; E2: gametogenic development; E3: morphological maturity; E4: spawning; and E5: postspawning).

The gonadal development of the grooved carpet shell clam was examined with the photos taken and classified, following a maturation six-stage scale proposed by Wilson & Seed [36], and modified by Ojea et al. [28]. Briefly, these stages are characterised as follows: sexual resting stage or E0, E1 stage or onset of gametogenesis, E2 or gametogenic development stage, E3 or morphological maturity stage, E4 or spawning stage, and E5 or post-spawning stage (Table 1 and Appendix A Figure A1).

### 2.6. Biochemical Composition

The biochemical composition of the gonadal tissues was analysed following standard methodologies. All analyses were carried out using gonad-visceral mass tissue previously frozen, lyophilized, and stored at −20 °C. Total protein composition was determined using the spectrophotometric method (spectrophotometer ONDA V-10 PLUS, ONDA Corporation, New Taipei City, Taiwan) proposed by Lowry [37] and modified by Nóvoa [38] with bovine serum albumin (Sigma-Aldrich, St. Louis, MO, USA) as standard; 2–3 mg of gonadal tissue was used, and results were read at 750 nm. Total carbohydrate quantity was determined following the anthrone (Merck, Darmstadt, Germany) spectrophotometric method proposed in 1956 by Fraga [39], using glycogen (Sigma-Aldrich, St. Louis, MO, USA) as standard and 2–3 mg of lyophilized tissue. Total lipid composition was analysed in the samples using 200 mg of lyophilized gonadal tissue and by the gravimetric chloroform-methanol (Supelco, Bellefonte, PA, USA) method proposed by Folch [40] and modified by Beninger and Lucas [41] and Pazos et al. [42]. Absorbance was read at 625 nm. Results were expressed as mg of each biochemical component per g of dry gonadal tissue.

### 2.7. Environmental Parameters

Water temperature and salinity were considered important environmental variables during the study period 2023–2025. Data were recorded by a nearby buoy from INTECMAR [43]. To compare these data with the previous study in the same location, Ojea et al.’s [28] data were used.

### 2.8. Statistical Analysis

Statistical analyses were carried out using the IBM SPSS statistical package (software IBM SPSS Version 29.0.2). To analyse the association between different variables (temperature, morphometric measurements, gametogenic development stage, biochemical composition, and condition indices), a Pearson correlation analysis was performed; to interpret results, r and *p*-value were analysed considering significant correlation with *p* < 0.05 or *p* < 0.01. Variables were previously analysed to confirm that statistical conditions met the requirements of normality and homogeneity of variance.

## 3. Results

### 3.1. Biometric Measurements

The biometric measurements are shown in Table 2. The mean length was 42.47 ± 2.08 mm, width 30.76 ± 1.66 mm, and thickness 20.26 ± 1.35 mm. The mean total fresh weight was 17.78 ± 2.96 g, the mean shell weight was 9.78 ± 1.65 g, the mean soft tissue weight was 3.85 ± 0.69 g, and the mean gonad tissue weight was 1.01 ± 0.33 g. Maximum values of each measurement were recorded in June 2023. The minimum values were slightly different: the measurement of length, width, and thickness reached a minimum in March 2024; fresh body weight was the lowest in May 2024; shell weight in June 2024, soft tissue weight in October 2024, and gonadal weight presented the lowest value in October 2024.

Pearson correlation analyses were carried out to evaluate patterns and relationships, or the absence of them, between sizes and weights. In Table 3, the results of these analyses are represented (r and *p*-value). There were three groups of correlations: very high significant correlations (r > 0.70, *p* < 0.001), these variables grew together and represented total size, including relationships between sizes (TSL, TSW, TST); sizes with FBW and SW, and FBW-SW and STW-GW. Moderate correlations were found between TSL-STW and STW-FBW (r ≈ 0.40–0.65, *p* < 0.001). Weak or non-significant correlations (*p* > 0.05) were mostly in STW with TSW, TST, and SW. GW had non-significant correlations with all variables except with STW (*p* < 0.001). This is proof that gonadal tissue growth was dependent of soft-tissue weight but independent of shell size, which is normal due to the seasonality of gonadal development.

### 3.2. Condition Indices

The results of condition indices were calculated using biometric measurements and are shown below in Table 4 with their individual standard deviation. IC values ranged from 16.7 to 30 (mean was 21.79 ± 3.59); BCI ranged from 30 to 60 (mean was 40.56 ± 8.21), and GCI, which was the most relevant in this study because it relates the gonadal development to the stage of the individuals, ranged from 19 to 36 (mean was 25.75 ± 12.01).

### 3.3. Sex Distribution

**Figure 1:**
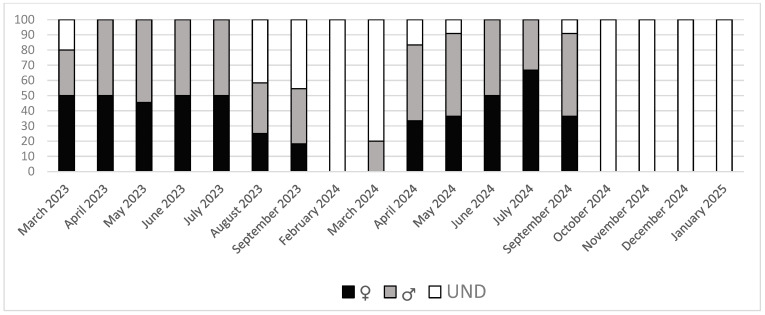
Sampling sex distribution of *R. decussatus* from the Baldaio lagoon (N.W. Spain) in percentage. Females (♀), males (♂), and undefined (UND).

The distribution of females and males in the sampling tended to be 50:50, but in some cases, this was not possible because of the immature stages of development, where sex could not be determined. The sampling of the monthly distribution of females, males, and undefined is shown in Figure 1. Two very different periods can be seen: a resting period between October and February and the gametogenesis period (including ripeness and spawning) between March and September.

### 3.4. Histological Study of Gametogenic Cycle

**Figure 2:**
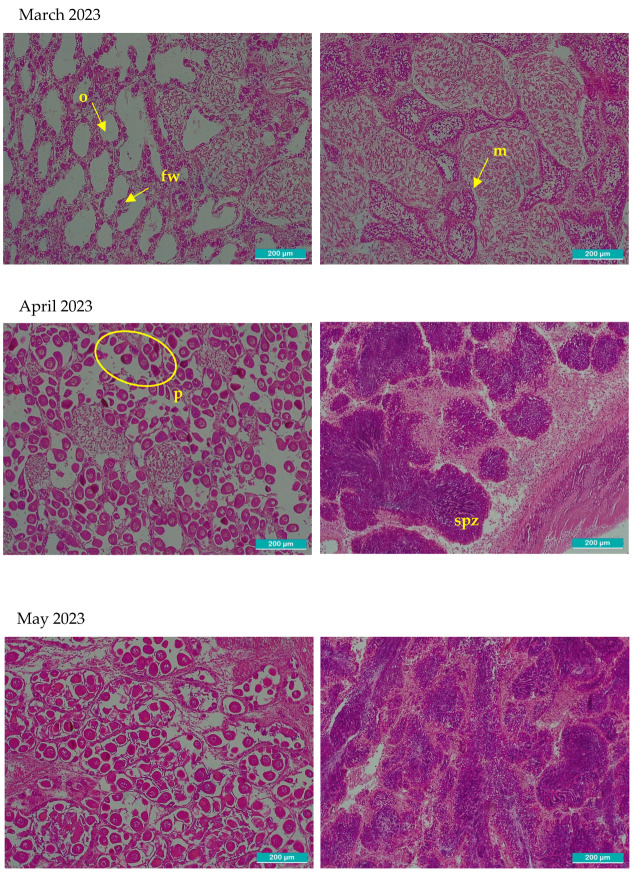

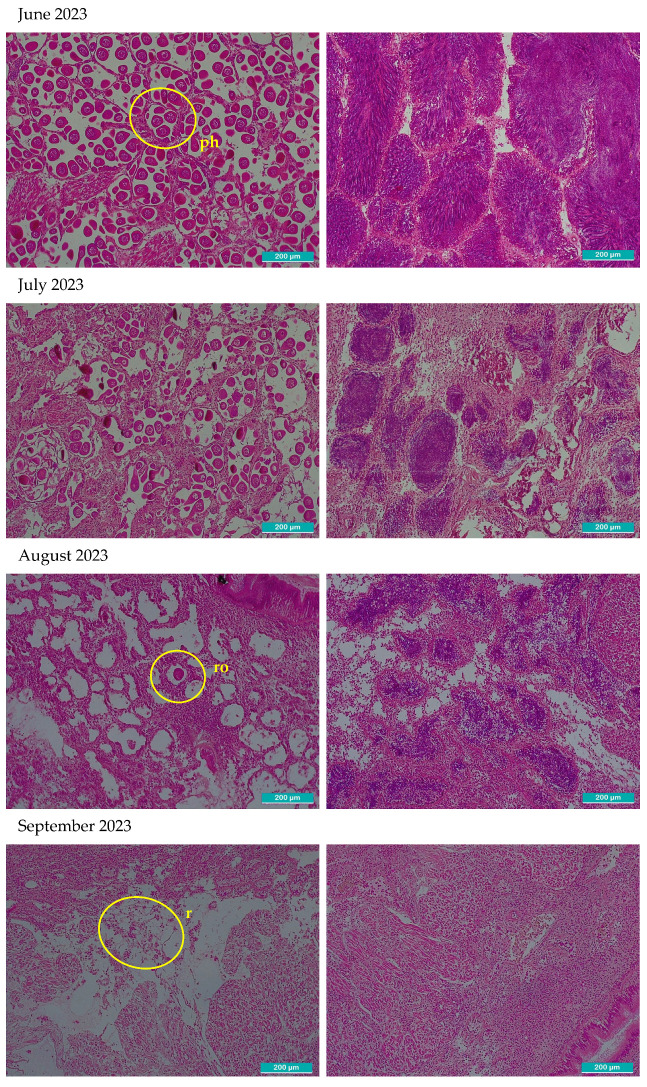

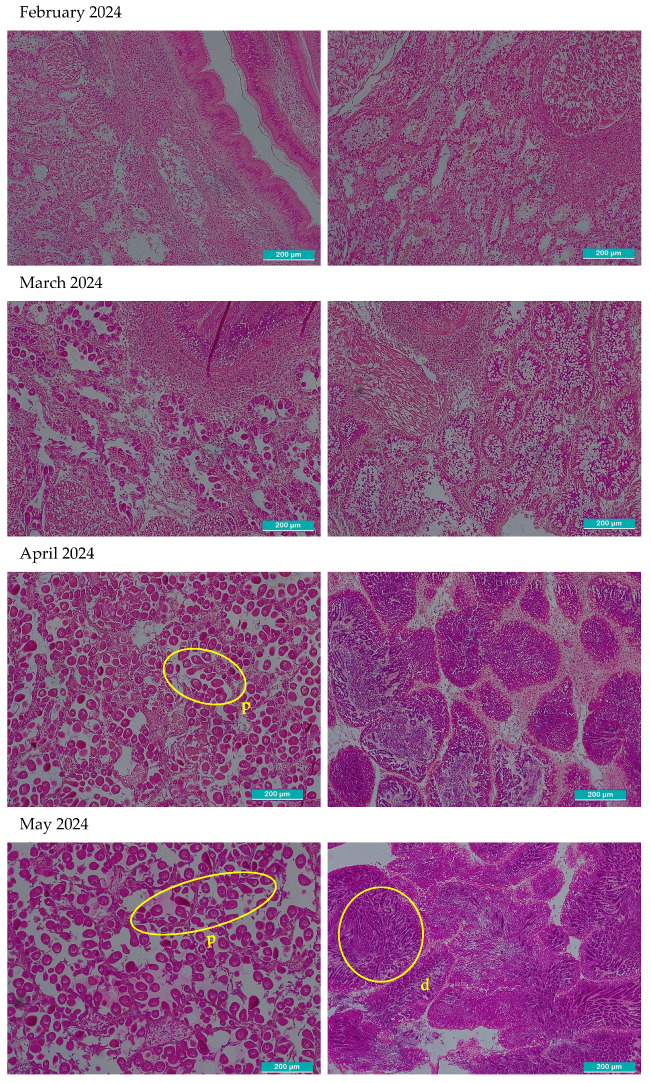

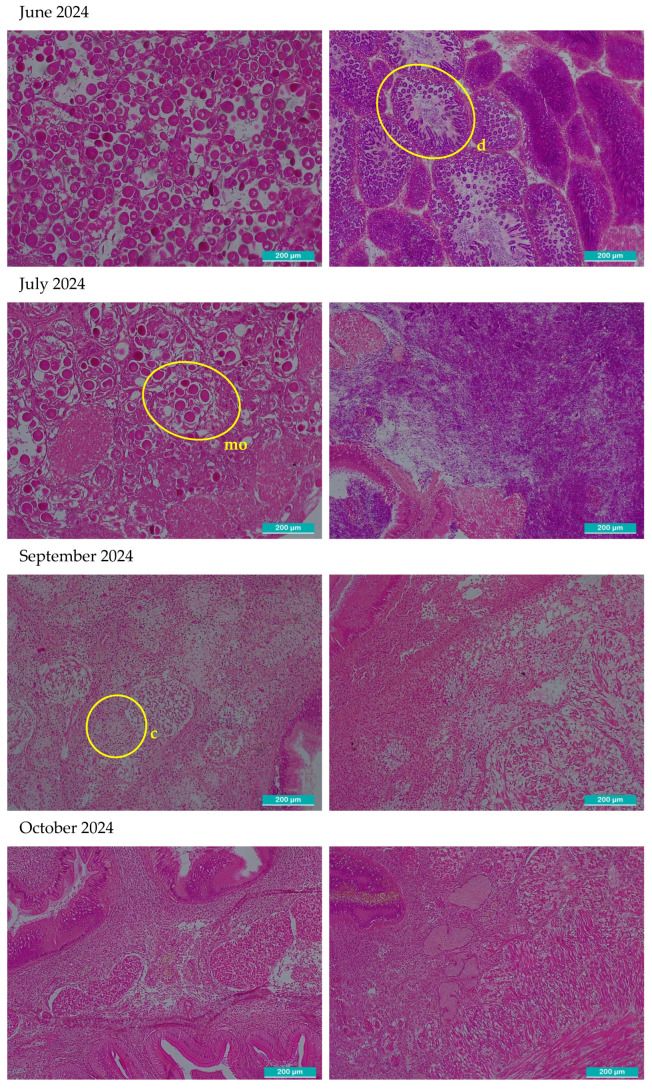

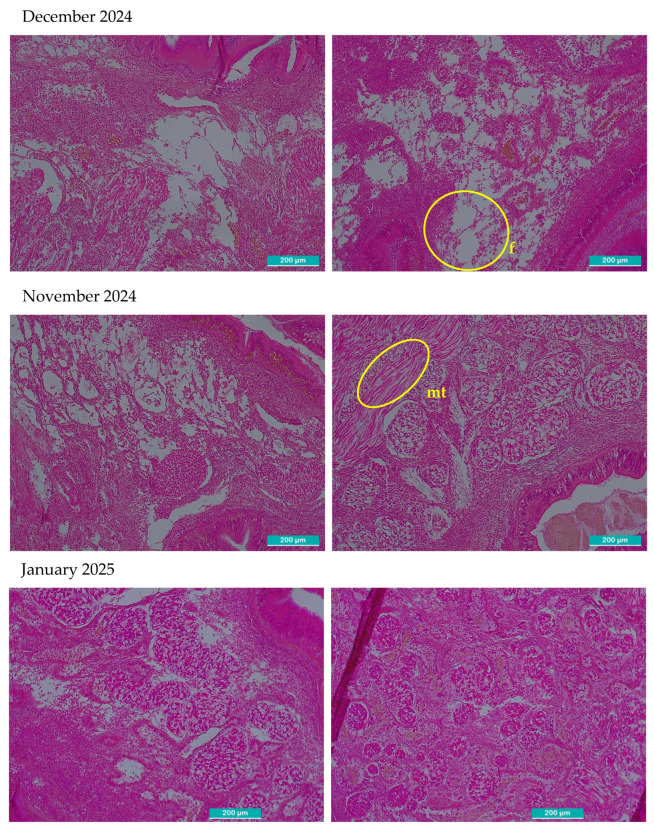
Microscopic histological images of sampled gonadal tissue of *R. decussatus* from Baldaio lagoon (N.W. Spain). Two 10x images per month are shown; the scale bar represents 200 µm. The left images correspond to female and the right to male gonadal tissue. Representative structures are marked. o: primordial oocytes in follicular wall; fw: follicular wall; m: male incipient follicle; p: pedunculated oocytes attached to the walls of the follicle; spz: spermatozoids; ph: polyhedral form of the ripe oocytes; ro: residual atretic oocyte; r: residuals from cytolysis after spawning d: radial arrangement of spermatogonia from the follicle periphery towards the lumen where mature spermatozoa accumulate and occasionally the gonadal duct; mo: mature oocyte in a gonad partially ripped; c: connective undifferentiated tissue; mt: muscular tissue; f: empty follicle.

After observation with the optical microscope, all principal characteristics of the development of the gonadal tissue were recorded, paying special attention to connective tissue, size and number of follicles, provision of the cells, and shape, and then each one was classified using the qualitative scale of gonadal development (Table 1). In Figure 2, two images per month are shown; the left image shows gonadal tissue from a female clam, and the right one is from a male. There were some months where sex could not be determined: September 2023, February, September–December 2024, and January 2025. In these cases, the two images included only connective tissue because follicles were not present.

### 3.5. Gametogenic Cycle and Maturation Scale Distribution

**Figure 3:**
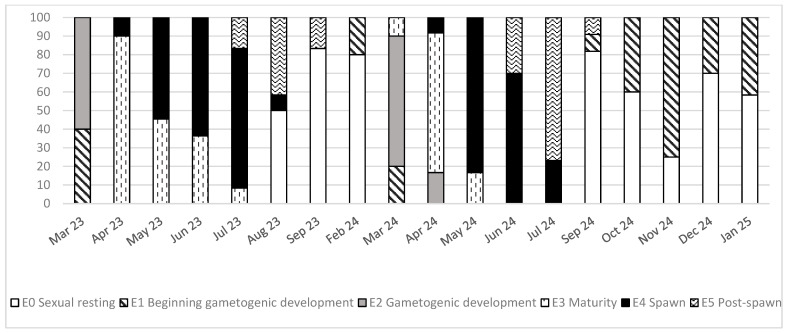
Monthly percentages of developmental stages of the gonads of *R. decussatus* from Baldaio lagoon (N.W. Spain).

The monthly percentage distribution of clams at each development stage is represented in Figure 3. When two stages were present in one sample, the predominant stage was considered for the classification of the sample.

In March, in the two sampling years, clams were in phase E1 (beginning of gametogenic development) or already E2 (gametogenic development); during April, May, and June of 2023, they were in E3 (maturity) and E4 (spawning) stages, and in July some clams were already in E5 (post-spawning stage), although most of them were still in E4 (spawning period). Later in September and the following months, all clams had already spawned, and the predominant stage was sexual resting. In 2024, there were some slight differences. In April, some clams were already E2 (gametogenic development), but the most relevant result was that the spawning period started in April, as in 2023, peaked in May and June, and by July, most of the clams were in the post-spawning stage. In September, E1 (beginning of gametogenic development) was present in some specimens, while in the previous year, there were no clams in this phase. In general, the main spawning activity was observed from April to August in both years, with peaks in May-June, and gametogenesis having begun in February-March. In 2024, the development of gonadal tissue started in September (Figure 3).

### 3.6. Biochemical Analyses

**Figure 4:**
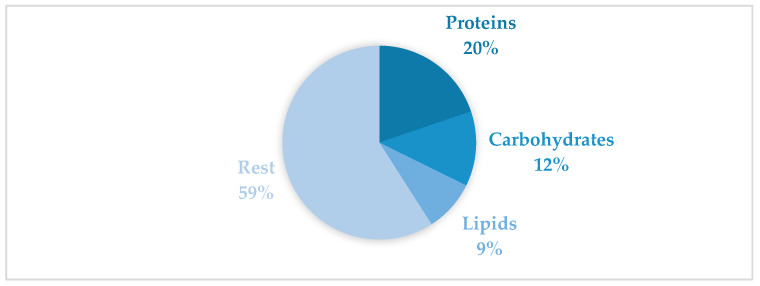
Percentages of mean biochemical composition in the gonadal tissue of *R. decussatus* from Baldaio lagoon (N.W. Spain).

The mean biochemical composition of the gonadal tissue is shown in Figure 4, represented as a schematic diagram of the gonadal tissue and the proportion of each biomolecule. Proteins represented 19.79% of the total, followed by carbohydrates at 12.49% and lipids, which were only 8.66%. The rest of the tissue consisted of non-solubilised proteins, mineral ashes, nucleic acids, vitamins, etc.

**Figure 5:**
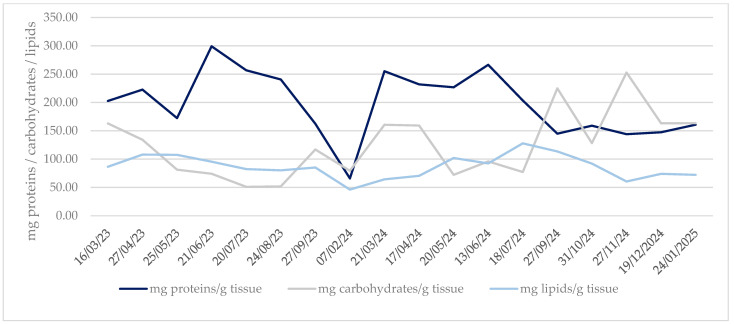
Evolution of biochemical composition of R. decussatus from Baldaio lagoon (N.W. Spain). Represented as mg of total proteins/ carbohydrates/lipids per g of gonadal tissue.

The monthly evolution of the biochemical composition of gonadal tissue along the sampling period is shown in Figure 5, where each biomolecule is expressed in mg/g tissue. Mean protein content was 197.90 mg protein/g tissue, with a maximum of 299.18 mg protein/g tissue in June 2023 and a minimum of 66.00 mg protein/g tissue in February 2024. These means of protein did not include non-solubilised proteins because the Lowry method only determines soluble proteins. Mean carbohydrate content was 124.95 mg carbohydrates/g tissue, the maximum was 252.77 mg carbohydrates/g tissue in November 2024, and the minimum was 51.08 mg carbohydrates/g tissue in July 2023. In the case of lipids, the mean was 86.66 mg lipids/g tissue, 127.85 mg lipids/g tissue was the maximum in July 2024, and 46.21 mg lipids/g tissue was the minimum in February 2024.

Biochemical composition was analysed in comparison with seawater temperature using Pearson correlation analyses. Results of these analyses are shown in Table 5. Correlation with carbohydrates was significant and negative, indicating that carbohydrates decreased with an increase in temperature. In the case of proteins and lipids, results showed no significant relationship with seawater temperature, *p*-value > 0.05.

Pearson correlation analysis was applied to evaluate the relation between condition indices and biochemical composition. Results shown in Table 6 indicated only a correlation between the gonadal condition index and protein and lipid concentration. CI and GCI had no correlation with protein, carbohydrate, and lipid concentration. GCI showed positive and significant correlations with proteins (r 0.517, *p* < 0.05) and lipids (r 0.644, *p* < 0.05) but not with carbohydrates (r −0.440, *p* > 0.05).

### 3.7. Temperature and Salinity in the Lagoon

**Figure 6:**
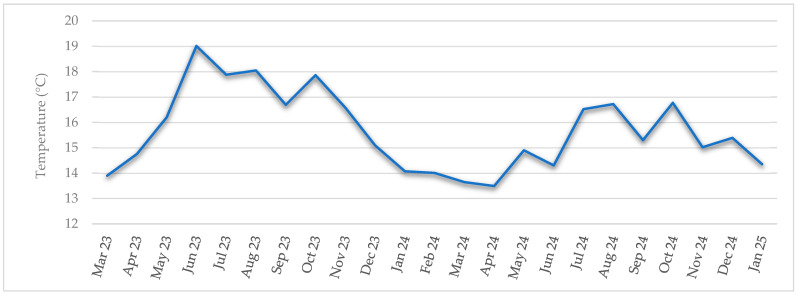
Monthly mean seawater temperature of the Baldaio lagoon (N.W. Spain) during the study period (°C).

Water temperature data from the Baldaio lagoon were obtained by processing raw buoy sensor data. The surface temperature ranged from 19.01 °C to 13.49 ± 1.58 °C, with a mean of 15.67 ± 1.58 °C. The maximum temperature was reached in June 2023, and the minimum in April 2024. Monthly mean temperature data are represented in Figure 6.

A series of Pearson correlation analyses was applied between each biometric variable and seawater temperature of the lagoon, paying attention to R and *p*-value. The results (Table 7) showed that in the cases of width and thickness measurements, water temperature had a significant positive effect, and moderate effects in width and thickness had the strongest effect, *p* < 0.01. Only in SW seawater temperature had a significant positive effect, *p*-value < 0.05. TBW, STW, and GW had a different behaviour; *p*-value > 0.05, temperature had no significant effect on these variables.

The effect of temperature on the condition indices of the clams was analysed using simple Pearson correlation analysis (Table 8). A significant negative relationship was observed between CI and temperature (*p*-value < 0.05), indicating that an increase in temperature is associated with a decrease in CI.

**Figure A2:**
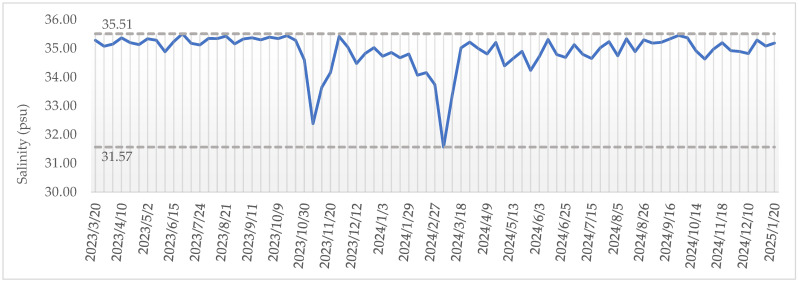
Salinity recorded in the Baldaio lagoon during the sampling period. The dashed grey lines show the salinity range including minimum and maximum values reached.

The mean monthly salinity in the lagoon ranged between 31.57 and 35.51 psu. (Appendix A, Figure A2).

## 4. Discussion

### 4.1. Effects of Lagoon Temperatures

The evolution of temperature inside the lagoon is slightly different from that of the open sea due to the special shape and oceanic dynamics of the lagoon, which is affected by rainstorms or strong land winds as well as by freshwater inputs, marine water inputs, and outputs. These dynamics had been previously described by Simantiris and Theocharis’ study in 2024 about the seasonal variability of hydrological parameters in a Mediterranean coastal lagoon, where they found that water circulation in the lagoon varied due to the interaction between freshwater and marine water inputs, but the system maintains a neutral annual balance [44]. Baldaio lagoon was selected as a sampling location because of its special abiotic characteristics and due to the availability of previous studies that allow for comparison and evaluation of the evolution over the past 20 years [28,45]. In the period 1998–1999, the temperature of the lagoon varied from 11.4 to 23.4 °C [28], but the temperatures registered in the current study ranged between 13.5 and 19.01 °C; this pattern is much smoother. High temperatures were not so high, and lower temperatures were neither so low. Minimum temperature varied from 11.4 °C in February 1999 to 13.49 °C in April 2024, and maximum temperature decreased from 23.4 °C in August 1999 to 19.01 °C in June 2023. The maximum temperature decreased by almost 4 °C, while the minimum mean temperature increased by approximately 2 °C. The annual mean temperature of the lagoon varied from 16.13 °C, 20 years ago, to 15.55 °C in the current study, a difference of nearly 1 °C. These results showed a different thermal pattern between the two analysed periods, 1998–1999 and 2023–2025. Although the mean temperature did not show a significant variation (16.13 → 15.67 °C ≈ 0.46 °C), a marked reduction in thermal amplitude was observed in minimums ≈ 2.1 °C (11.4 → 13.49 °C) and maximums ≈ 4.4 °C (23.4 → 19.01 °C). Although global warming is usually associated with higher thermal means, local dynamics could have some differences and result in a reduced range of temperatures. The increase in minimum temperatures coincides with climate patterns of southern Europe, where global warming is causing milder winters. However, the decrease in maximum temperatures could be related to other oceanographic factors, like recent evidence of a slowdown in the Atlantic Overturning Meridional Circulation (AMOC), which has been widely documented in recent years [46,47,48]. McCarthy et al. [46] described the variability and mechanisms of AMOC, showing evidence that its intensity has changed in recent decades. Smeed et al. [47] provided strong observational evidence that the AMOC is a major factor in decadal-scale variability of the North Atlantic climate. And Caesar et al. [48] documented a weakening of the AMOC by about 3 ± 1 Sverdrups (around 15 percent) since the mid-twentieth century. This weakened AMOC reduces heat transport to the northeastern Atlantic and decreases the thermal contrast between warm surface waters and cold deep waters. There is no data to indicate whether the depth of the lagoon may influence this fact. As a result, the probability of cooler water inputs into coastal areas, like the studied lagoon, increases during the summer months, which could explain the decrease in maximum temperatures observed in the recent period.

The recorded temperatures in the lagoon reflected a variability that has significant biological implications. For example, gametogenesis, as mentioned above [7,9,11,49], is highly temperature-dependent in *R. decussatus* or condition indices and biochemical parameters. Furthermore, the stock population of *R. decussatus* can be affected because larvae and juveniles are highly sensitive to temperature and salinity variations, increasing their mortality and directly affecting the recruitment and finally the stock population [50]. However, to be able to confirm that these changes are due to global or regional climate change processes, a longer time sampling would be necessary. Actually, using a simulation model based on Dynamic Energy Budget theory, Maynou et al. [51] predicted that with an increase in temperature of 3 °C, the growth and reproductive capacity of *R. philippinarum* and *R. decussatus* would be affected. Thermal comfort areas for various species of bivalves could be affected in the future, according to the scenario obtained by the three-dimensional hydrodynamic model (Delft3D); the findings indicated that the internal part of the Galician Rías Baixas (N.W. Spain) could be negatively affected by the increase in seawater temperature by the end of the 21st century [52].

### 4.2. Biometrics Measurements

Temporal patterns revealed a remarkable seasonal growing dynamic of *R. decussatus*. FBW and STW showed the most pronounced variations, with maximums during high-temperature months, especially June 2023, which agrees with data reported in other bivalve species, where raising temperature and abundance of food stimulated growth and biomineralization [23,50,53]. Posterior decreasing of FBW suggested a response to less favourable environmental conditions. In contrast, STW showed stable values, indicating that the body of the animal kept a physiological range necessary to perform basic functions even under fluctuating environmental conditions and could be maintained by the protective effect of the shell. GW presented slightly increasing values, which repeated during spring and at the end of the year, reflecting a seasonal reproductive cycle synchronised with environmental factors such as temperature and nutrient availability. Morphometric measurements confirm that patterns and length showed high seasonal variability, while width and thickness were more stable, suggesting a higher sensitivity of longitudinal dimensions to environmental changes. Similar results were found by Ojea et al. [28] in their study in the same lagoon. When they analysed seasonal variation in weight and biochemical composition of the tissues in relation to the gametogenic cycle, they found that variations in body and gonad growth may occur in response to changes in environmental conditions. Whole data indicated that *R. decussatus* adjusted its own growth and reproduction in response to seasonality and environmental conditions, which may be relevant to the management and conservation of the population.

### 4.3. Seasonal Variations in Gametogenic Cycle

Gonadal development evolution in the *R. decussatus* clams of the Baldaio lagoon showed a seasonal pattern related to water temperature; the spawning period, which is the highest stage of gonadal development, occurred between April–August 2023 and April–July 2024, having peaks in May–June, coincident with peaks in seawater temperature (May 2023 and July 2024), suggesting a direct relationship between gonadal maturation and the temperature in the lagoon (Figure 3 and Figure 6). Other authors described the relationship between temperature and gonadal development in bivalves, such as Maneiro et al. [27,54], reporting a difference of 4 weeks in the beginning of the spawning period as a result of the combined effect of temperature and photoperiod in *O. edulis* compared with the control group at low temperature. Previous studies demonstrated that temperature has a direct influence on reproductive processes because of its relationship with gametogenesis [20,23,26]. Toba et al. [55] found that in Manila clams (*R. philippinarum*), gonadal development rate increased proportionally with temperature in a range between 10 and 27 °C. Velez et al. [49] showed that high temperatures of 28 °C could inhibit gonadal development in tropical mussels, and Delgado et al. [23] revealed species-specific variations in the reproductive behaviour *of R. decussatus* and *R. philipinarum* related to temperature. Especially interesting is the comparison of the present results with previous data reported by Ojea et al. [28] in the same species and lagoon, which stated that the spawning period lasted from May to August, with a peak in July-August. The comparison with the results presented in the current study showed that, in 20 years, the spawning pattern of *R. decussatus* in Baldaio lagoon changed both in the onset and in its duration. Twenty years ago, the onset began in May, but in our study, it occurred in April, and the end was in August, while before it was in September, even in October. The spawning peak was in July or August 20 years ago, but in the current study, it was in June-July; all this shows a difference in the onset of the spawning period, in the start of the resting period, and in the peak of the spawning occurred one month earlier. Previous authors confirmed the findings of Ojea et al. that the spawning period of *R. decussatus* occurred in summer [22]. The missing data due to weather conditions or logistical circumstances were not expected to provide much information, because the clams were mainly in a sexual resting period. There are several ecological implications to this finding. On the one hand, if the spawning period is brought forward, larvae could have difficulties surviving because they are not adapted to different conditions [56]. On the other hand, temperature is the main factor affecting the metabolism of the clams; Papadopoulos et al. [15] showed that with higher temperatures, clams promote lipid oxidation and increased metabolism connected to a delay in the activation of anaerobic pathways. Furthermore, abnormal mortalities of *R. decussatus* were reported, linked to changes in temperature and salinity [30]. These environmental conditions may also affect other communities that inhabit the lagoon, which, in some cases, are directly related to the feeding of clams, such as phytoplankton and zooplankton. These aspects mentioned above could explain, along with others, the decline of the population.

### 4.4. Condition Indices

Variations in condition indices were observed during all the years; CI and BCI showed similar patterns; they are indices that give us an idea of the stage of the clam’s nutritional and reproductive condition; in both cases, they were maximum during springtime of 2024 and minimum during summer of 2023, which is related to the stage of gonadal development and temperature, as in Manila clam [55]. During the spawning period recorded in May 2024, almost all the clams sampled were in the E4 stage. At this time, the nutrition and gonadal condition of the clams are the highest because of the abundance of nutrients and food provided by upwelling events very frequent during spring and summer. In addition, in July of 2023, some animals had recently spawned, and this could be the reason for such low condition index values. The gonadal condition index was the most relevant in this study because of its direct relationship with gonadal development; the minimum value was registered in October, instead of summer, when gonadal tissue was depleted during the resting period, although the maximum value was in May 2024, following the same pattern as the other indices.

### 4.5. Biochemical Composition

In the gonadal tissue of *R. decussatus* clams from the Baldaio lagoon, biochemical composition showed a clear seasonal pattern throughout the study period. Proteins were the most abundant biomolecules present in the tissue, with high peaks in early summer (June and July 2023) and spring 2024, followed by decreases during winter, which reflect periods of intense metabolic demand and possibly reduced feeding activity. Carbohydrates had more irregular patterns with sudden falls during spring and autumn, suggesting short-term energy storage events possibly related to food availability, recovery after spawning, or thermal-salinity stress events. Lipid fractions were low and stable during all years except for a peak during late summer 2024. These patterns showed that biochemical composition varied in response to environmental conditions and the reproductive cycle of gonadal development, as it was stated by Pérez Camacho et al. [10]. A significant correlation between GCI and protein and lipid concentration is consistent and agrees with the findings of Ojea et al. [45], where the protein content and total lipid content were directly related to GCI, but not with the findings about total glycogen content (carbohydrates). However, other studies showed an important role of carbohydrates in the reproduction of bivalves with maximum glycogen content immediately before and during gamete development [57,58], which is consistent with the current data. Similar results were found by Li et al. [59], who suggested that carbohydrates played the most important role in reproduction in the clam *Mactra chinensis* (Philippi, 1846), with lipid biosynthesis linked to glycogen breakdown and protein content with a synchronous increase with oocyte size and lipid concentration.

## 5. Conclusions

The present work studied the possible effect of seawater temperature under climate change on a population of *R. decussatus* clams of the Galician coast (N.W. Spain). The histological analysis of gonadal development showed a one-month advance in the spawning period of *R. decussatus* clams of the Baldaio lagoon compared with 20 years ago, starting in April instead of May; and in fact, the entire annual cycle of gonadal development-resting phase has suffered changes.

## Figures and Tables

**Table 1:** Description of the gonadal qualitative scale of development of *R. decussatus*.

Phases	Characteristics	Microscopic Elements
Phase E0	Sexual resting stage	Connective tissue fills the gonad; in the absence of gametes, sexual identification is not possible.
Phase E1	Onset of gametogenesis	Germinal cells are present; the initial formation of follicles is very difficult to determine the sex of individuals, especially in the early phase of the stage.
Phase E2	Gametogenic development	Follicles fill a large part of the tissue, oocytes in females have grown, and spermatocytes fill the follicles; sex can be identified in almost all the specimens.
Phase E3	Morphological maturity	Gonad filled with mature oocytes or spermatozoa arranged in sheets towards the centre of the follicles; in females, the oocytes reach their largest size, and the pressure on the follicles makes them polyhedral in shape.
Phase E4	Spawning	In females, oocytes detach from the follicle walls, and in males, spermatozoa lose their radial arrangement.
Phase E5	Postspawning	Follicles disintegrate, and only remnants of gametes are present; the animal enters a sexual resting phase, and follicle walls are broken.

When two or more phases are present in the same sample, the predominant stage was considered for the classification of the sample.

**Table 2:** Monthly mean (±SD) of clam shell and tissue measurements and weights (mm, g) of *R. decussatus* from the Baldaio lagoon.

	TSL (mm)	TSW (mm)	TST (mm)	FBW (g)	SW (g)	STW (g)	GW (g)
	M ± SD	M ± SD	M ± SD	M ± SD	M ± SD	M ± SD	M ± SD
March 2023	45.38 ± 2.36	32.43 ± 1.72	21.65 ± 1.16	21.79 ± 3.14	11.43 ± 1.19	4.35 ± 0.48	0.96 ± 0.14
April 2023	43.05 ± 1.24	31.12 ± 1.01	21.33 ± 0.99	18.37 ± 1.88	10.05 ± 1.1	4.56 ± 0.46	1.29 ± 0.24
May 2023	44.39 ± 3.26	31.94 ± 1.98	21.63 ± 1.16	20.9 ± 3.23	11.89 ± 1.56	4.86 ± 0.7	1.37 ± 0.28
June 2023	**47.8 ± 2.45**	**34.24 ± 1.49**	**22.89 ± 1.04**	**25.21 ± 3.45**	**13.34 ± 1.46**	**5.26 ± 0.87**	**1.63 ± 0.33**
July 2023	42.83 ± 0.98	31.23 ± 0.9	21.46 ± 0.77	19.4 ± 1.61	11.05 ± 1.19	3.26 ± 0.64	1.03 ± 0.33
August 2023	42.41 ± 0.85	31.13 ± 0.79	22 ± 2.55	18.38 ± 1.85	10.39 ± 0.96	3.42 ± 0.58	0.68 ± 0.14
September 2023	42.37 ± 1.3	34.08 ± 1.94	19.86 ± 1.05	16.82 ± 2.45	8.88 ± 1.62	3.24 ± 0.39	0.75 ± 0.17
February 2024	42.27 ± 1.4	30.22 ± 0.77	19.77 ± 0.75	17.72 ± 1.68	9.68 ± 1.22	3.59 ± 0.26	0.73 ± 0.06
March 2024	**38.58 ± 2.01**	**27.92 ± 1.49**	**18.26 ± 1.24**	14.43 ± 2.56	7.78 ± 1.59	3.09 ± 0.47	0.65 ± 0.12
April 2024	41.55 ± 0.92	30.08 ± 0.85	19.13 ± 0.75	16.24 ± 1.25	8.6 ± 0.93	4.19 ± 0.54	1.23 ± 0.27
May 2024	40.15 ± 2.57	28.95 ± 1.67	18.19 ± 1.24	13.48 ± 2.87	7.69 ± 1.66	4.51 ± 0.6	1.61 ± 0.31
June 2024	41.11 ± 0.82	28.8 ± 1.14	18.48 ± 0.81	**13.54 ± 1.39**	**6.92 ± 0.89**	3.72 ± 0.4	1.09 ± 0.2
July 2024	40.63 ± 1.86	29.4 ± 1.4	19.64 ± 2.43	14.86 ± 2.13	8.27 ± 1.43	3.15 ± 0.51	1.12 ± 0.26
September 2024	42.79 ± 2.46	30.83 ± 1.36	20.53 ± 1.51	18.17 ± 3.3	9.54 ± 1.82	4.48 ± 0.62	1.16 ± 0.27
October 2024	41.19 ± 2.5	29.35 ± 1.82	19.85 ± 1.41	16.72 ± 3.38	10.04 ± 1.63	**2.95 ± 0.58**	**0.59 ± 0.16**
November 2024	41.26 ± 2.25	30.02 ± 1.75	19.87 ± 1.58	16.56 ± 3.18	9.16 ± 1.95	3.32 ± 0.75	0.71 ± 0.18
December 2024	42.72 ± 1.21	30.77 ± 0.86	20.63 ± 0.77	19.35 ± 1.62	11.37 ± 1.36	3.51 ± 0.59	0.75 ± 0.23
January 2025	44 ± 1.87	31.18 ± 1.38	19.59 ± 1.88	18.01 ± 3.18	9.88 ± 2.09	3.81 ± 0.5	0.81 ± 0.15

Values highlighted in bold represent the maximum and minimum of the study period.

**Table 3:** Statistical results of Pearson analyses of morphometric measurements of *R. decussatus* from the Baldaio lagoon.

		TSW	TST	FBW	SW	STW	GW
TSL	R	0.844 **	0.823 **	0.933 **	0.858 **	0.648 *	0.365
	*p*-value	<0.001	<0.001	<0.001	<0.001	0.004	0.137
	N	18	18	18	18	18	18
TSW	R		0.745 **	0.795 **	0.714 **	0.433	0.214
	*p*-value		<0.001	<0.001	<0.001	0.073	0.395
	N		18	18	18	18	18
TST	R			0.908 **	0.906 **	0.417	0.192
	*p*-value			<0.001	<0.001	0.085	0.445
	N			18	18	18	18
FBW	R				0.966 **	0.537 *	0.234
	*p*-value				<0.001	0.022	0.35
	N				18	18	18
SW	R					0.433	0.161
	*p*-value					0.072	0.524
	N					18	18
STW	R						0.833 **
	*p*-value						<0.001
	N						18

Significant correlation: * at 0.05 level (two-tailed), ** 0.01 level (two-tailed), non-significant correlation > 0.05.

**Table 4:** Condition index (IC), dry condition index (BCI), and gonadal condition index (GCI) of *R. decussatus* from the Baldaio lagoon (monthly mean ± SD).

	IC	BCI	GCI
	M ± SD	M ± SD	M ± SD
March 2023	20.93 ± 1.63	38.21 ± 3.51	22.17 ± 2.16
April 2023	25.92 ± 2.11	45.53 ± 3.21	28.13 ± 3.19
May 2023	22.43 ± 2.42	41.14 ± 5.95	28.23 ± 3.94
June 2023	21.7 ± 1.99	39.39 ± 4.35	31.18 ± 5.19
July 2023	**16.7 ± 2.73**	**29.55 ± 5.29**	31.3 ± 6.57
August 2023	18.31 ± 3.11	33.11 ± 5.93	19.98 ± 2.41
September 2023	19.53 ± 3.63	37.56 ± 8	23.1 ± 4.08
February 2024	20.29 ± 0.85	37.29 ± 2.44	20.43 ± 1.38
March 2024	21.57 ± 1.6	40.31 ± 4.09	20.98 ± 2.16
April 2024	25.7 ± 3.53	49.36 ± 8.57	29.07 ± 3.69
May 2024	**29.84 ± 1.66**	**59.53 ± 5.55**	**35.46 ± 2.85**
June 2024	26.54 ± 1.84	54.12 ± 5.43	29.25 ± 3.34
July 2024	20.8 ± 2.72	38.56 ± 7.06	35.32 ± 4.13
September 2024	25.21 ± 2.47	47.62 ± 5.61	25.55 ± 3.34
October 2024	17 ± 2.75	29.69 ± 5.02	**19.71 ± 2.03**
November 2024	20.25 ± 3.84	36.95 ± 8.09	21.37 ± 2.25
December 2024	18.01 ± 1.78	30.89 ± 3.71	20.99 ± 3.44
January 2025	21.54 ± 3.78	39.7 ± 7.82	21.26 ± 3.29

Values highlighted in bold are maximums and minimums.

**Table 5:** Pearson correlation analyses between the mean temperature of the Baldaio lagoon and the biochemical composition of the gonadal tissue of *R. decussatus*.

		Proteins	Carbohydrates	Lipids
Temperature	R	0.302	**−** **0** **.515**	0.340
	*p*-value	0.224	**0** **.029**	0.168
	N	18	**18**	18

Data highlighted in bold showed significant correlation (*p* < 0.05).

**Table 6:** Pearson correlation analysis between condition indices and biochemical composition of *R. decussatus* from the Baldaio lagoon.

Index	Biomolecule	r	*p*-Value	N
CI	Proteins	0.246	0.326	18
	Carbohydrates	0.081	0.750	18
	Lipids	0.333	0.176	18
BCI	Proteins	0.259	0.300	18
	Carbohydrates	0.040	0.875	18
	Lipids	0.298	0.230	18
GCI	Proteins	**0** **.517**	**0** **.028**	**18**
	Carbohydrates	−0.440	0.067	18
	Lipids	**0** **.644**	**0** **.004**	**18**

Data highlighted in bold showed significant correlation, *p* < 0.05.

**Table 7:** Pearson correlation analysis between the mean temperature of the Baldaio lagoon and biometric measurements of *R. decussatus*.

	Temperature	
Length	R	0.403
	*p*-value	0.098
	N	18
Width	R	0.507 *
	*p*-value	**0.032**
	N	**18**
Thickness	R	0.637 **
	*p*-value	**0.004**
	N	**18**
TBW	R	0.469
	*p*-value	0.05
	N	18
SW	R	0.535 *
	*p*-value	**0.022**
	N	**18**
STW	R	0.018
	*p*-value	0.945
	N	18
GW	R	0.148
	*p*-value	0.557
	N	18

Data highlighted in bold showed significant correlation, * *p* < 0.05, ** *p* < 0.01.

**Table 8:** Pearson correlation analysis between the mean temperature of the Baldaio lagoon and condition indices of *R. decussatus*.

	Temperature	
CI	R	**−0.476**
	*p*-value	**0.046**
	N	**18**
BCI	R	−0.468
	*p*-value	0.050
	N	18
GCI	R	0.204
	*p*-value	0.417
	N	18

Data highlighted in bold showed significant correlation, *p* < 0.05.

## Data Availability

The original contributions presented in this study are included in the article. Further inquiries can be directed to the corresponding authors.

## Abbreviations

BCI	Body condition index
CI	Condition index
CIMA	Centro de Investigacións Mariñas de Galicia
DCI	Dry condition index
FBW	Total fresh body weight
GCI	Gonadal condition index
GW	Gonadal-hepatopancreas tissue weight
SD	Standard deviation
STW	Fresh soft tissue weight
SW	Shell weight
TSL	Total shell length
TST	Total shell thickness
TSW	Total shell width

## Appendix A. Additional Methodological Information

### Appendix A.1. Sampling Effort and Sample Allocation

**Table A1:** Number of individuals collected from Baldaio lagoon and analysed per sampling period.

Sampling Period	Total Collected (n)	Biometry (n)	Histology (n)
March 2023	20	20	10
April 2023	20	20	10
May 2023	18	18	11
June 2023	20	20	11
July 2023	20	20	12
August 2023	20	20	12
September 2023	23	23	12
February 2024	5	5	5
March 2024	20	20	10
April 2024	20	20	12
May 2024	20	20	11
June 2024	20	20	10
July 2024	20	20	13
September 2024	20	20	11
October 2024	16	16	10
November 2024	9	9	9
December 2024	20	20	10
January 2025	12	12	12
